# Moderators of antidepressant augmentation versus switch in the OPTIMUM randomised controlled trial

**DOI:** 10.1192/bjp.2025.125

**Published:** 2025-11

**Authors:** Helena K. Kim, Jordan F. Karp, Helen Lavretsky, Daniel M. Blumberger, Patrick J. Brown, Alastair J. Flint, Emily Lenard, J. Philip Miller, Charles F. Reynolds, Steven P. Roose, Eric J. Lenze, Benoit H. Mulsant

**Affiliations:** Department of Psychiatry, Temerty Faculty of Medicine, University of Toronto, Toronto, Canada; Department of Psychiatry, University of Arizona, Tucson, USA; Semel Institute for Neuroscience and Human Behavior, University of California, Los Angeles, USA; Campbell Family Mental Health Research Institute, Centre for Addiction and Mental Health, Toronto, Canada; Department of Geriatric Psychiatry, Program on Healthy Aging and Late Life Brain Disorders, New York State Psychiatric Institute, Columbia University Medical Center, New York, USA; Centre for Mental Health, University Health Network, Toronto, Canada; Department of Psychiatry, Healthy Mind Lab, School of Medicine, Washington University in St Louis, St Louis, USA; Institute of Informatics, Data Science and Biostatistics, School of Medicine, Washington University in St Louis, St Louis, USA; Department of Psychiatry, University of Pittsburgh School of Medicine, Pittsburgh, USA

**Keywords:** Treatment-resistant depression, late-life depression, antidepressant, augmentation, moderator

## Abstract

**Background:**

Older adults with treatment-resistant depression (TRD) benefit more from treatment augmentation than switching. It is useful to identify moderators that influence these treatment strategies for personalised medicine.

**Aims:**

Our objective was to test whether age, executive dysfunction, comorbid medical burden, comorbid anxiety or the number of previous adequate antidepressant trials could moderate the superiority of augmentation over switching. A significant moderator would influence the differential effect of augmentation versus switching on treatment outcomes.

**Method:**

We performed a preplanned moderation analysis of data from the Optimizing Outcomes of Treatment-Resistant Depression in Older Adults (OPTIMUM) randomised controlled trial (*N* = 742). Participants were 60 years old or older with TRD. Participants were either (a) randomised to antidepressant augmentation with aripiprazole (2.5–15 mg), bupropion (150–450 mg) or lithium (target serum drug level 0.6 mmol/L) or (b) switched to bupropion (150–450 mg) or nortriptyline (target serum drug level 80–120 ng/mL). Treatment duration was 10 weeks. The two main outcomes of this analysis were (a) symptom improvement, defined as change in Montgomery–Asberg Depression Rating Scale (MADRS) scores from baseline to week 10 and (b) remission, defined as MADRS score of 10 or less at week 10.

**Results:**

Of the 742 participants, 480 were randomised to augmentation and 262 to switching. The number of adequate previous antidepressant trials was a significant moderator of depression symptom improvement (*b* = −1.6, *t* = −2.1, *P* = 0.033, 95% CI [−3.0, −0.1], where *b* is the coefficient of the relationship (i.e. effect size), and *t* is the *t*-statistic for that coefficient associated with the *P*-value). The effect was similar across all augmentation strategies. No other putative moderators were significant.

**Conclusions:**

Augmenting was superior to switching antidepressants only in older patients with fewer than three previous antidepressant trials. This suggests that other intervention strategies should be considered following three or more trials.

Treatment-resistant depression (TRD) is typically defined as a major depressive disorder that does not remit after at least two antidepressant trials of adequate dosage and duration.^1^ Common pharmacotherapy strategies for patients with TRD include augmentation with another medication (which could be a second antidepressant or a psychotropic medication from another class – for example, an atypical antipsychotic or a mood stabiliser) or switching to a different antidepressant. The Optimizing Outcomes of Treatment-Resistant Depression in Older Adults (OPTIMUM) clinical trial compared these two strategies in older adults with TRD.^2,3^ We recently reported that augmentation with aripiprazole is superior to switching to bupropion,^3^ consistent with a growing body of literature favouring augmentation over switching strategies in the treatment of TRD.^4,5^ In this context, the TRD field has converged on comparing these two strategies rather than looking at specific medications.^4^

Identifying moderators that influence the effectiveness of these treatment strategies advances personalised medicine. For example, a moderator that can be assessed by a clinician, such as age, helps the clinician choose the optimal treatment strategy based on individual characteristics in day-to-day practice. Moderators can also expand our understanding of the biological mechanisms involved in a disorder or its treatment. To date, five factors have consistently been shown to negatively influence treatment outcomes with antidepressant treatment in late life: age;^6^ executive dysfunction^7^; comorbid medical burden;^8,9^ comorbid anxiety;^6,7^ and degree of treatment resistance.^10,11^ Therefore, the second aim of the OPTIMUM study was to assess whether these five factors moderate the effect of augmentation versus switching. A preplanned moderation analysis was performed to test the hypothesis that these five factors will moderate symptom improvement and depression remission in older adults with TRD. Baseline depression severity, which can bias the interpretation of factors associated with treatment outcomes,^12^ was added as a covariate in the moderation analysis, as done in previous analyses of clinical trials.^13–15^

## Method

### Study overview

OPTIMUM was a pragmatic, open-label, randomised clinical trial (NCT02960763) that received approval by the institutional review boards of the five sites where the trial was conducted (Columbia University; University of California, Los Angeles; University of Pittsburgh; University of Toronto; and Washington University in St. Louis). Its design,^2^ sample size calculation, recruitment, follow-up^3^ and primary report^3^ have been published previously.

### Ethics statement

The authors assert that all procedures contributing to this work comply with the ethical standards of the relevant national and institutional committees on human experimentation, and with the Helsinki Declaration of 1975 as revised in 2013. All procedures involving human subjects/patients were approved by the institutional review boards (IRBs) at each of the five trial sites (IRB ID nos: Centre for Addiction and Mental Health, 086/2016; New York State Psychiatric Institute/Columbia University, 7409; University of Pittsburgh, PRO16100179; University of California, Los Angeles, 16-001829; Washington University in St. Louis, 201609085).

### Randomisation

**Fig. 1:**
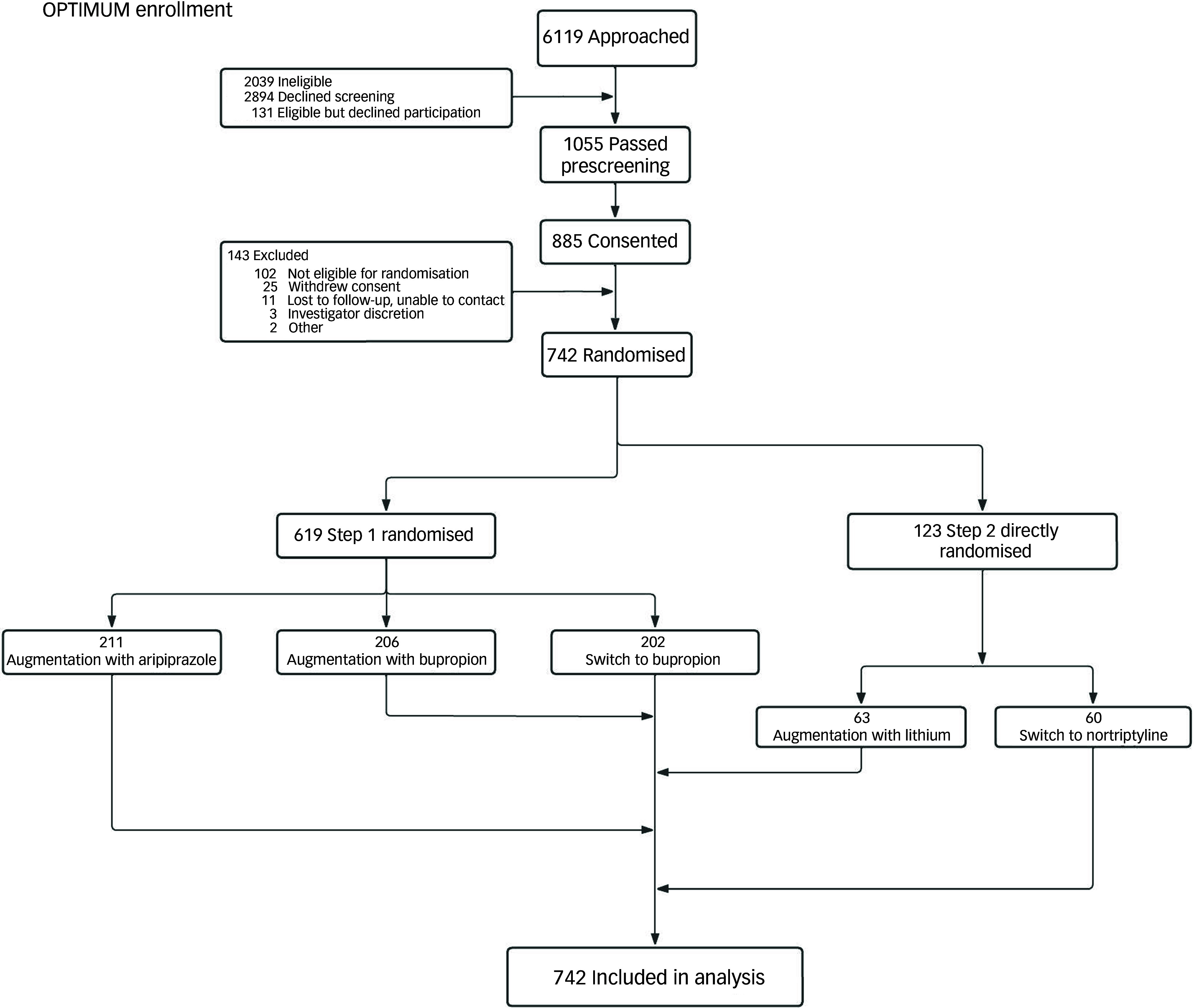
Enrollment, randomisation and inclusion in analysis from steps 1 and 2. OPTIMUM, Optimizing Outcomes of Treatment-Resistant Depression in Older Adults.

Details of randomisation have been published previously.^3^ Briefly, in step 1 (duration, 10 weeks) of the trial, participants were randomly assigned in a 1:1:1 ratio to augmenting their current antidepressant with aripiprazole (2.5–15 mg daily) or bupropion (150–450 mg daily) or switching to bupropion (150–450 mg). If the participant had already tried one of the step 1 medications without clinical benefit or tolerability, they entered step 2 directly without going through step 1 (duration, 10 weeks) and were randomised in a 1:1 ratio to either (a) augmentation of their current antidepressant with lithium (targeting a serum drug level of 0.6 mmol/L) or (b) a switch to nortriptyline (targeting a serum drug level of 80–120 ng/mL). In this preplanned moderation analysis, we used data from participants in step 1 and from those who entered directly into step 2 (Fig. 1). We did not include participants who were entered into step 2 after not attaining remission in step 1. This is because those who entered step 2 after not attaining remission in step 1 would have had 10 additional weeks of treatment in a clinical trial setting compared with those who were in step 1, or compared with those who had direct entry into step 2. This approach permitted us to analyse baseline and week 10 data (i.e. 10 weeks after they had started their randomised pharmacotherapy).

### Participant characteristics

Participants were 60 years of age or older with TRD, defined as not remitting after two or more antidepressant trials of adequate dosage and duration at any time during the current episode. TRD was ascertained using the Antidepressant Treatment History Form (ATHF; Buchalter et al^11^). Race was self-reported by the participants. All participants gave written informed consent after being fully informed about the study details.

### Statistical analysis

The published primary report of the OPTIMUM results showed the superiority of aripiprazole augmentation compared with switching to bupropion for change in depression severity and remission.^3^ Because the sample size was adjusted from 1500 to 708 mid-study, a post hoc calculation showed that a sample size of 561 would provide sufficient power (0.8, *α* = 0.05) to detect a small effect size (0.14) in a regression model consisting of 3 variables (treatment arm, moderator and covariate). There is robust evidence supporting the superiority of augmentation over switch strategies, with the TRD field converging on comparing these two strategies rather than looking at the specific medications.^4,5^ Therefore, our statistical analysis plan specified that this preplanned analysis would focus on comparing the overall strategy of augmentation versus switch on the same two depression clinical outcomes as the primary report (i.e. change in depression severity and remission). The five *a priori* hypothesised moderators were age, executive dysfunction, comorbid medical burden, comorbid anxiety and degree of treatment resistance. Executive dysfunction was measured using the US National Institutes of Health (NIH) Toolbox fluid cognition composite, with a score of 85 or lower indicating impairment.^16^ Comorbid medical burden was measured using the Cumulative Illness Rating Scale – Geriatric (CIRS-G).^17^ Comorbid anxiety was measured using the Patient Reported Outcomes Measurement Information System (PROMIS) anxiety scale, with a score of 60 or higher (one standard deviation above average anxiety) being considered clinically anxious.^18^ The degree of treatment resistance was defined as the number of previous adequate antidepressant trials during the past 2 years, or during the current episode if the episode had lasted less than 2 years. The number of previous adequate antidepressant trials during the past 2 years (or current episode if the episode had lasted less than 2 years) was obtained from ATHF.^19^ Only antidepressants prescribed during either the duration of the current major depressive episode or the past 2 years, whichever was shorter, were considered when rating ATHF, because of concerns over the accuracy of recalling specific antidepressants taken years before (and even more so, their dosages and duration). Executive dysfunction and comorbid anxiety were dichotomised because the scales used to measure these hypothesised moderators have thresholds for determining clinically significant impairment, allowing for clinical application of the findings. Based on the results of the primary report,^3^ we anticipated that approximately 15% of participants included in this analysis would not have week 10 MADRS scores. The two outcomes were change in MADRS scores (i.e. depression symptom improvement) and remission. We used the same definition of remission as in the published primary report,^3^ which was defined as MADRS^20^ score ≤10 at week 10 (of step 1 or 2). MADRS <10 was reported to be concordant with remission as defined by the Clinical Global Impression Scale for Severity.^21^ We performed Little’s missing completely at random (MCAR) test:^22^ as expected with an effectiveness clinical trial, the outcome data were missing not at random (MNAR; *χ* ^2^ = 1115.9, d.f. = 75, *P* = 0.002). Thus, we did not impute MNAR missing outcome data.

IBM SPSS 27 for Windows was used for all statistical analyses, with averages expressed as mean ± standard deviation. Analysis of covariance (ANCOVA) was used for comparing change in MADRS scores between the two treatment strategies. Log binomial regression was used for comparing remission rates between the two treatment strategies, because it carries a lower risk of inflating effect sizes and produces more readily interpretable outcomes than logistic regression.^23^ The results are expressed in prevalence ratios and confidence intervals. Residual plots were used to check for homoscedasticity (i.e. assumption of equal variance). The PROCESS Macro extension^24^ was used to perform moderation analysis through previously reported methods.^25^ Moderation analysis was performed with listwise deletion to handle missing data as per the design of the programme. Briefly, moderation analysis using PROCESS performs a regression analysis with the independent variable (i.e. treatment strategies), the potential moderator (e.g. age) and the interaction term between the independent variable and the potential moderator predicting the dependent variable (e.g. change in MADRS scores). Bootstrapping is performed in PROCESS to mitigate non-normal distribution of data;. A moderation effect is found if the interaction term has a significant effect on the dependent variable. Conditional effects of the independent variable on the dependent variable are also given at different levels of the moderator, to illustrate the moderating effect. Continuous variables that define products were mean centred. Simple slope analysis was performed for moderators identified as being significant, with each potential moderator being analysed separately. Baseline MADRS score was included as a covariate in all analyses. A Spearman’s rank correlation analysis was performed among the five potential moderators and baseline MADRS scores. Age (rho = −0.10, *n* = 717, *P* = 0.008), comorbid anxiety (rho = 0.3, *n* = 623, *P* < 0.001) and number of previous adequate antidepressant trials (rho = 0.08, *n* = 717, *P* = 0.042) were all significantly correlated with baseline MADRS scores, supporting the addition of baseline MADRS as a covariate. A further Spearman’s rank correlation analysis was performed among the five potential moderators to identify potential confounders. The only two significant correlations found were between age and CIRS-G score (rho = 0.13, *P* < 0.001) and between age and executive dysfunction (rho = 0.31, *P* < 0.001). Subsequently, moderation analysis for age included CIRS-G score and executive dysfunction as covariates; moderation analysis for both CIRS-G and executive dysfunction included age as a covariate. Corrections for multiple comparisons were not performed, because this preplanned analysis assessed a small set of prespecified potential moderators.^26^ We used the previously published minimal clinically important difference (MCID) in MADRS scores of 1.6–1.9^27^ to interpret the findings.

## Results

### Participant characteristics

Most of the participants were from step 1 and, by design, around two-thirds received augmentation while the other third were switched to a different agent (Fig. 1). Participant characteristics are shown in Table 1. Of 742 participants, 480 (64.7%) received augmentation (of whom 415 [86.5%] had week 10 MADRS scores) and 262 (35.3%) were switched to a different treatment (of whom 215 [82.1%] had week 10 MADRS scores; total discontinuation rate, 15.1%). Because some data were missing for three moderator variables, the following number (%) of participants had complete data for CIRS-G score: 476 (99.2%) in augmentation, 261 (99.6%) in switch; PROMIS anxiety score: 408 (85.0%) in augmentation, 227 (86.6%) in switch; and fluid cognition score, 295 (61.5%) in augmentation, 168 (69.4%) in switch.

**Table 1:** Participant characteristics

Parameter	Augmentation	Switch	Total^a^ (mean ± s.d. or *N* (%))
Sample size (*N*)	480	262	742
Steps	Step 1, 417 (86.9%)	Step 1, 202 (77.1%)	Step 1, 619 (83.4%)
	Step 2, 63 (13.1%)	Step 2, 60 (22.9%)	Step 2, 123 (16.6%)
Treatment groups			Augmentation with aripiprazole, 211 (28.4%) Augmentation with bupropion, 206 (27.8%) Switch to bupropion, 202 (27.2%) Augmentation with lithium, 63 (8.5%) Switch to nortriptyline, 60 (8.1%)
Treatment strategy			Augmentation, 480 (64.7%) Switch, 262 (35.3%)
Age (years)	68.9 ± 6.6	69.3 ± 7.3	69.1 ± 6.8
Self-reported gender	Women, 333 (69.4%) Men, 147 (30.6%)	Women, 167 (63.7%) Men, 95 (36.3%)	Women, 500 (67.4%) Men, 242 (32.6%)
Race	Caucasian, 408 (85.0%) Other, 72 (15.0%)	Caucasian, 230 (87.8%) Other, 32 (12.2%)	Caucasian, 638 (86.0%) Other, 104 (14.0%)
Years of education	14.6 ± 2.9 (*n* = 471)	15.0 ± 2.8 (*n* = 257)	14.7 ± 2.9 (*n* = 728)
CIRS-G score	8.7 ± 4.7 (*n* = 476)	8.6 ± 4.6 (*n* = 261)	8.7 ± 4.7 (*n* = 737)
Number of previous adequate antidepressant trials (ATHF score)	2.3 ± 0.8 (*n* = 480)	2.4 ± 0.9 (*n* = 262)	2.4 ± 0.8 (*n* = 742)
PROMIS anxiety score	63.6 ± 7.2 (*n* = 408)	64.1 ± 7.7 (*n* = 227)	63.8 ± 7.4 (*n* = 635)
Clinical anxiety	Yes, 303 (74.3%) No, 105 (25.7%)	Yes, 168 (74.0%) No, 59 (26.0%)	Yes, 471 (74.2%) No, 164 (25.8%)
Fluid cognition score	87.0 ± 10.6 (*n* = 295)	86.6 ± 11.2 (*n* = 168)	86.8 ± 10.8 (*n* = 463)
Executive dysfunction	Yes, 140 (47.5%) No, 155 (52.5%)	Yes, 73 (43.5%) No, 95 (56.5%)	Yes, 213 (46.0%) No, 250 (54.0%)
MADRS score at baseline	23.3 ± 7.3 (*n* = 464)	23.0 ± 7.0 (*n* = 253)	23.2 ± 7.2 (*n* = 717)
MADRS score at week 10	16.0 ± 9.2 (*n* = 415)	18.1 ± 9.4 (*n* = 215)	16.7 ± 9.3 (*n* = 630)
Change in MADRS score	7.1 ± 8.0 (*n* = 415)	4.7 ± 9.1 (*n* = 212)	6.3 ± 8.5 (*n* = 627)
Remission at week 10^b^	Remission, 130 (31.3%) No remission, 285 (68.7%)	Remission, 51 (23.7%) No remission, 164 (76.3%)	Remission, 181 (28.7%) No remission, 449 (71.3%)

ATHF, Antidepressant Treatment History Form; CIRS-G, Cumulative Illness Rating Scale – Geriatric; MADRS, Montgomery–Asberg Depression Rating Scale; PROMIS, Patient-Reported Outcomes Measurement Information System.

a. Total sample size is 742 unless indicated otherwise.

b. Remission defined as having a MADRS score of 10 or less at week 10.

### Moderation analyses

We combined steps 1 and 2 for our analysis.^3^ Consistent with the original report, our sample showed a larger mean (s.d.) change in MADRS score with augmentation (−7.1 ± 8.0) than switching (−4.7 ± 9.1). There was also a significant difference in remission rates between the two treatment strategies, where 130 (31.3%) remitted with augmentation versus 51 (23.7%) with switch (prevalence ratio 1.1, 95% CI [1.0, 1.2], d.f = 1, *P* = 0.035).

The mean (s.d.) number of adequate previous antidepressant trials was 2.4 (0.8). The number of adequate previous antidepressant trials had a significant negative moderating effect on the change in depression scores (*b* = −1.55, *t* = −2.13, *P* = 0.033, 95%CI [−2.98, −0.12]), where *b* is the coefficient of the relationship (i.e. effect size) and *t* is the *t*-statistic for that coefficient associated with the *P*-value. Examining conditional effects of the focal predictor (i.e. treatment strategies) on the outcome (i.e. change in MADRS score) at different levels of the moderator (i.e. number of previous antidepressant trials) with the Johnson−Neyman technique, using the average as the centre (average number of previous antidepressant trials, 2.4) and comparing those with 1 s.d. below the average versus 1 s.d. above the average,^28^ augmentation was superior to switching in participants with 1 s.d. fewer trials compared with the average (effect 3.62, *P* = 0.0001). This superiority of augmentation over switching was diminished in participants with 1 s.d. more previous adequate trials compared with the average (effect 0.88, *P* = 0.337), i.e. those with two previous trials versus three. Simple slope analysis showed that the impact of previous adequate antidepressant trials on the decrease in MADRS scores was larger with augmentation than with switching.

**Fig. 2:**
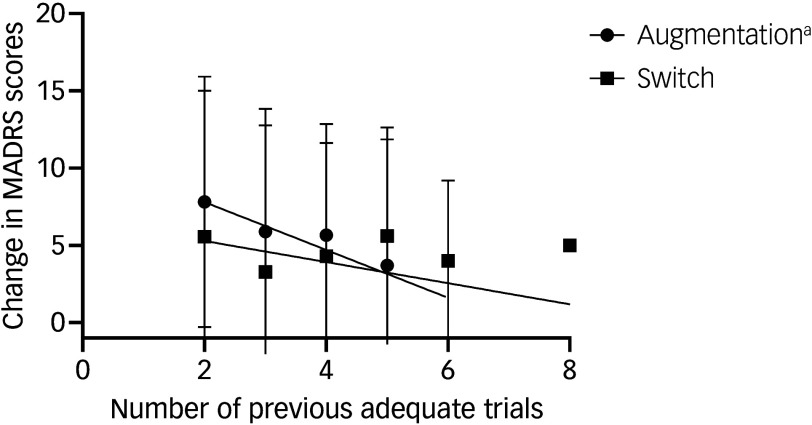
Simple linear regression analysis of change in Montgomery–Åsberg Depression Rating Scale (MADRS) scores versus number of previous adequate antidepressant trials in the augmentation group (*n* = 415) and the switch group (*n* = 212). Error bars represent standard deviation. a. In the augmentation group, change in MADRS scores was significantly associated with the number of previous adequate trials (*y* = −1.5*x* + 10.9; *F*_1, 392_ = 8.5, *P* = 0.004). In the switch group, change in MADRS scores was not associated with the number of previous adequate trials (*y* = −0.7*x* + 6.7; *F*_1, 201_ = 1.1, *P* = 0.301), where *y* indicates the outcome variable (i.e. change in MADRS scores), *x* indicates the independent variable (i.e. number of previous adequate trials), *F* indicates the *F*-statistic, which is used to derive the significance of the regression model, and ‘1, 392’ following *F* indicates the degrees of freedom.

We examined post hoc whether the number of previous adequate trials was a moderator of the change in MADRS scores, expecting that the exposure−response relationship between these two variables would be stronger in the augmentation group compared with the switch group. Thus, we performed post hoc simple linear regression analysis between change in MADRS scores versus the number of previous adequate antidepressant trials in the augmentation and switch groups, including participants with two or more trials (Fig. 2). In the augmentation group, there was a significant association between the change in MADRS scores and the number of previous adequate trials (*y* = −1.5*x* + 10.9; *F*_1, 392_ = 8.5, *P* = 0.004, where *y* indicates the outcome variable (i.e. change in MADRS scores), *x* indicates the independent variable (i.e. number of previous adequate trials), *F* indicates the *F*-statistic, which is used to derive the significance of the regression model, and ‘1, 392’ following *F* indicates the degrees of freedom). In the switch group, there was no association between change in MADRS scores and the number of previous adequate trials (*y* = −0.7*x* + 6.7; *F*_1, 201_ = 1.1, *P* = 0.301).

We also performed post hoc moderation analysis to examine whether the moderating effect on the different treatment strategies was driven by the different medications used in each treatment strategy (i.e. aripiprazole, bupropion and lithium were used for augmentation versus bupropion and nortriptyline used for switching). There was no moderating effect of the number of previous adequate antidepressant trials on the relationship between the medication used (aripiprazole versus bupropion) and change in MADRS scores in step 1 (*b* = −0.2, *t* = −0.2, *P* = 0.839, 95% CI [−1.9, 1.6]). In step 2, lithium and nortriptyline did not significantly differ in regard to their effect on MADRS scores, negating the utility of moderation analysis.

Previous adequate antidepressant trials are classified into augmentation or monotherapy. A further post hoc moderation analysis was performed to examine whether there was a moderating effect of a previous antidepressant trial being an augmentation trial or not. Of the 742 participants, 75 (10.1%) had at least one previous augmentation trial prior to enrolling, with 8 (1.1%) having two previous augmentation trials. There was no moderating effect of whether a participant had a past augmentation trial on the relationship between treatment strategies used and change in MADRS scores (*b* = −2.7, *t* = −1.2, *P* = 0.214, 95% CI [−7.1, 1.6]).

Age (*b* = 0.2, *t* = 1.4, *P* = 0.161, 95% CI [−0.1, 0.4], executive dysfunction (*b* = 1.5, *t* = 0.9, *P* = 0.374, 95% CI [−1.8, 4.7]), comorbid medical burden (*b* = 0.1, *t* = 0.7, *P* = 0.497, 95% CI [−0.2, 0.4]) and comorbid anxiety (*b* = −0.40, *t* = −0.24, *P* = 0.810, 95% CI [−3.67, 2.87]) did not have a moderating effect on the relationship between treatment strategy and change in MADRS scores.

Similarly, none of these five characteristics had a moderating effect on the relationship between treatment strategy and remission rates (age: *b* = 0.1, *z* = 1.5, *P* = 0.123, 95% CI [−0.02, 0.1]; executive dysfunction: *b* = 0.3, *z* = 0.7, *P* = 0.485, 95% CI [−0.6, 1.3]; comorbid medical burden: *b* = 0.1, *z* = 1.4, *P* = 0.153, 95% CI [−0.02, 0.2]; comorbid anxiety: *b* = -0.42, *z* = −0.87, *P* = 0.38, 95% CI [−1.38, 0.53]; and degree of treatment resistance: *b* = −0.26, *z* = −1.10, *P* = 0.27, 95% CI [−0.72, 0.20]).

Sensitivity analyses showed that being of very old age (75 years or older) also did not moderate the relationship between treatment strategy and change in MADRS scores (*b* = 1.1, *t* = 0.5, *P* = 0.615, 95% CI [−3.2, 5.3]) or remission (*b* = 0.7, *z* = 1.1, *P* = 0.281, 95% CI [−0.6, 1.9]). Similarly, very impaired executive function (NIH Toolbox Fluid Cognition composite score 70 or lower) did not moderate the relationship between treatment strategy and change in MADRS scores (*b* = 0.01, *t* = 0.003, *P* = 0.997, 95% CI [−5.8, 5.8]) or remission (*b* = 1.2, *z* = 1.3, *P* = 0.201, 95% CI [−0.6, 3.0]).

## Discussion

We analysed data from the OPTIMUM randomised clinical trial comparing augmentation versus switching strategies in older adults with TRD. We assessed five hypothesised moderators (age, executive dysfunction, comorbid medical burden, comorbid anxiety and degree of treatment resistance) on the relationship between the two strategies and treatment outcomes, as measured by change in MADRS scores or remission at week 10. Only the number of previous adequate antidepressant trials (i.e. degree of treatment resistance) was a significant moderator, where higher number of trials decreased one’s likelihood of benefitting from augmentation over switching. Specifically, our findings suggest that the benefit of augmentation over switching is diminished in patients with three or more previous adequate antidepressant trials. We did not find a moderating effect of age, executive dysfunction, comorbid medical burden or comorbid anxiety, suggesting that these patient-level characteristics are less likely to impact whether someone will benefit from augmentation over switching.

Previous studies have reported that having had an adequate antidepressant trial predicts poor subsequent treatment outcomes in older adults.^10,11^ Our results suggest that the number of previous antidepressant trials is also an important consideration, because those with fewer than three may benefit more from augmenting rather than switching. A meta-analysis reported the superiority of antipsychotic augmentation compared with ongoing antidepressant monotherapy, with an increasing number of up to four previous antidepressant trials.^29^ Our analysis shows that, compared with switching, the effect of augmentation on symptom improvement decreases with increasing number of antidepressant trials (up to eight). This moderating effect appears to be specific to the treatment strategy and is not limited to the specific medications used. It also appears to be independent of whether a previous antidepressant trial was an augmentation trial or not. It has been consistently shown that treatment failure begets more treatment failures in depression.^1,11,30^ This may be due to psychological factors, such as worsening treatment expectations,^31^ or biological factors, such as progressive neurobiological changes or more severe immune alterations in those with TRD versus those without.^32,33^ In a Cochrane review, augmentation strategies showed modest benefit compared with switching in patients with TRD.^4^ Our results suggest that this modest benefit may disappear with a higher degree of treatment resistance.

We did not identify any moderators for remission. Because less than a third of participants attained remission, optimising the reduction of symptom severity by selecting the optimal treatment strategy is an important clinical goal in older adults with TRD. Our findings also indicate that augmentation and switching strategies perform both similarly and poorly in patients with multiple previous adequate antidepressant trials. This suggests that older patients with TRD with more than three adequate antidepressant trials should be considered for other intervention strategies, such as ketamine^34^ or brain stimulation,^35^ recognising that treatment response rates with these strategies decrease with treatment resistance as well.

Previous studies have reported age and executive dysfunction as moderators of treatment outcome to aripiprazole augmentation.^6,7^ We did not find this using two different methods to examine age (either as a continuous variable or setting a threshold for ‘very old’), and using two different thresholds for executive dysfunction (impaired or very impaired). One possible explanation is that our participants were required to have had at least two failed previous antidepressant trials to be included in the study, while those other studies required only one previous trial. This may have resulted in the lack of effect of previously identified moderators in our more treatment-resistant sample. Furthermore, the study reporting age as a moderator examined a wider range of ages, from <50 to >65 years;^6^ our sample consisted only of older adults with a smaller age range. Our previous study examined executive dysfunction as a moderator of treatment outcome in late-life depression used the Trail Making test;^7^ in that study we used the Fluid cognition score from the NIH Toolbox, which is a broad measure of fluid cognitive ability that allows for the separation of those with executive dysfunction and those without.^16^ A meta-analysis showed that planning and organisation, but not cognitive flexibility, were associated with poor antidepressant treatment outcome in late-life depression,^36^ suggesting that different domains of executive dysfunction may vary in their ability to act as moderators. Our previous studies have also reported comorbid physical illness and anxiety as negative predictors of treatment outcome in late-life depression.^8,9,37^ While a predictor can also be a moderator, that is not necessarily the case: it depends on the sample and relationships being examined.^38^ Our sample also consisted of older out-patients, typically with mild to moderate burden of comorbid physical illness. Having an unstable physical illness (e.g. an unmanaged cardiovascular condition) was also an exclusion criterion, which may have impacted our findings. Future studies are needed to validate these findings in older adults with a higher burden of physical illness. Notwithstanding the need for further exploration, our results suggest that, in older adults with TRD without severe executive dysfunction or medical comorbidities, neither their age, executive dysfunction, physical impairment nor level of anxiety pose additional barriers to benefiting from augmentation pharmacotherapy.

### Strengths and limitations

Our analysis has some strengths and limitations. The OPTIMUM study is the largest clinical trial to date in older adults with TRD.^3^ It was a randomised clinical trial with standardised treatments focusing on broad strategies of augmentation versus switch, as opposed to specific medications, consistent with a growing consensus in the TRD field.^4,5,39^ To our knowledge, this analysis is also the first to compare the effect of augmentation versus switch on depression symptom improvement in relation to the number of previous antidepressant trials. This preplanned analysis also had *a priori* focus on a predetermined list of five potential moderators selected based on previous studies reporting their association with treatment outcomes. Limitations include the retrospective collection of medication history, although collateral sources of information were used (e.g. health or pharmacy records) when available. Only antidepressants prescribed for up to 2 years were considered when rating the ATHF, which is a potential limitation given that some participants had major depressive episodes that lasted longer than 2 years. Furthermore, the version of the ATHF we used does not capture why medications were discontinued (e.g. lack of tolerability or lack of efficacy). Future studies should explore whether reasons for discontinuing antidepressant medications moderate the outcomes of future treatment. We used the same MADRS threshold of 10 for remission as the primary OPTIMUM analysis, which is a potential limitation because it may not indicate an absence of depression. In addition, 15.1% of participants did not have week 10 MADRS scores due to some having dropped out.^3^ While the proportion of missing data was similar between switch and augmentation strategies, we cannot rule out the possibility of bias being introduced due to missing data. For example, it is reasonable to assume that participants who dropped out prior to week 10 had less improvement in their symptoms, influencing the strength of the interaction between previous antidepressant trials and treatment strategies. While recent TRD studies have compared augmentation versus switching rather than focusing on specific antidepressants,^39^ some moderators may have differential effects on specific antidepressants when their therapeutic or adverse effects are directly related to them. For example, one study reported that mirtazapine’s superiority over selective serotonin reuptake inhibitors was moderated by differences in sleep and appetite, which are two prominent adverse effects of mirtazapine.^40^ While our analysis showed that the moderating effect of the number of previous antidepressant trials is not due to the specific medication used, future studies could also look at the effect of other putative moderators on specific medications. Finally, our sample had limited diversity compared with the US or Canadian population, and participants were treated with four agents. Replicating this analysis in more varied samples with different agents will be important.

In conclusion, the findings of the OPTIMUM study can inform physicians and future studies on the selection of optimal treatment strategies for older patients with TRD. These findings identified readily available factors (i.e. the number of previous adequate antidepressant trials) that can be used to personalise clinical decision-making. Specifically, older patients with TRD with fewer than three previous adequate antidepressant trials may benefit more from augmentation strategies than switching to a different medication. Future studies are needed to identify effective treatments in patients with more than three previous adequate antidepressant trials.

## Data Availability

The data for this study are available upon request following submission and approval of a data request proposal. Please contact the corresponding author, B.H.M.
